# Tongue microbiota in relation to the breathing preference in children undergoing orthodontic treatment

**DOI:** 10.1186/s12903-024-05062-3

**Published:** 2024-10-21

**Authors:** Zuzana Marincak Vrankova, Petra Brenerova, Lenka Bodokyova, Jan Bohm, Filip Ruzicka, Petra Borilova Linhartova

**Affiliations:** 1 RECETOX, Faculty of Science, Masaryk University, Kotlarska 2, Brno, Czech Republic; 2 Clinic of Maxillofacial Surgery, University Hospital Brno, Jihlavska 20, Brno, Czech Republic; 3 Clinic of Stomatology, Institution Shared With St. Anne´s University Hospital, Faculty of Medicine, Masaryk University, Pekarska 53, Brno, Czech Republic; 4 Clinic of Microbiology, Institution Shared With St. Anne ´s University Hospital, Faculty of Medicine, Masaryk University, Pekarska 53, Brno, Czech Republic

**Keywords:** Tongue microbiota, Mouth breathing, Orthodontic treatment, Children, Pediatric sleep apnea, Craniofacial anomaly, Halitosis, *Solobacterium*, Oral candida

## Abstract

**Background:**

Mouth breathing (MB), a risk factor of oral dysbiosis and halitosis, is linked with craniofacial anomalies and pediatric obstructive sleep apnea. Here, we aimed to analyze tongue microbiota in children from the perspective of their breathing pattern before/during orthodontic treatment.

**Methods:**

This prospective case–control study included 30 children with orthodontic anomalies, 15 with MB and 15 with nasal breathing (NB), matched by age, sex, and body mass index. All underwent orthodontic examination and sleep apnea monitoring. Tongue swabs were collected before starting (timepoint M0) and approx. six months into the orthodontic therapy (timepoint M6). Oral candidas and bacteriome were analyzed using mass spectrometry technique and 16S rRNA sequencing, respectively.

**Results:**

MB was associated with higher apnea–hypopnea index. At M0, oral candidas were equally present in both groups. At M6, *Candida* sp. were found in six children with MB but in none with NB. No significant differences in bacterial diversity were observed between groups and timepoints. However, presence/relative abundance of genus *Solobacterium* was higher in children with MB than NB at M0.

**Conclusions:**

Significant links between MB and the presence of genus *Solobacterium* (M0) as well as *Candida* sp. (M6) were found in children with orthodontic anomalies, highlighting the risk of halitosis in them.

**Supplementary Information:**

The online version contains supplementary material available at 10.1186/s12903-024-05062-3.

## Introduction

The importance of physiological breathing, particularly nasal breathing (NB), is a frequently discussed topic. According to the literature, NB supports the natural development of the craniofacial region by promoting proper posture of the tongue against the palate, aiding in the expansion of the maxillary arch and proper alignment of the teeth [1]. In contrast, mouth breathing preference (MB) often results from nasal obstruction or habitual factors and can have significant consequences on craniofacial growth and development, potentially leading to a cascade of structural and functional changes throughout the body [2–4].

In children, MB can lead to an insufficient lip seal, an open mouth posture, and a series of craniofacial alterations such as a narrow, constricted hard palate, hyperdivergent growth patterns of the mandible, and elongation of the lower third of the face [2, 3, 5]. These alterations are not merely cosmetic but have functional implications, including an increased risk of developing dental malocclusions, such as overjet and crowding, and of pediatric obstructive sleep apnea (POSA) [6, 7]. The altered craniofacial structure associated with MB may result in the narrowing of the upper airways, increasing the likelihood of airway collapse during sleep, which is a hallmark of POSA [6, 7].

The reduced salivary flow and increased mouth dryness, common in mouth breathers, create an environment conducive to the growth of microorganisms with pathogenic potential [8]. This imbalance often results in oral health issues such as dental caries, periodontal diseases, and halitosis [4]. Halitosis, or bad breath, is multifactorial disease particularly common among mouth breathers and is associated with tongue coating, *Candida* species, and putrefactive actions of anaerobic bacteria that thrive in the environment of a dry mouth [9, 10]. The altered oral microbiota in mouth breathers not only affects oral health but may also have systemic implications [4].

Systemically, chronic MB can affect overall health by altering respiratory patterns, reducing the quality of sleep, and impacting posture. The forward head posture commonly adopted by mouth breathers to open the airway further contributes to postural imbalances, which can lead to musculoskeletal issues over time [11]. Additionally, the continuous use of accessory muscles for breathing, instead of diaphragmatic breathing, may affect oxygenation and overall physical development in children [12].

This complex interplay between the MB, oral dysbiosis and associated oral diseases, orthodontic anomalies, orthodontic therapy, and POSA calls for a comprehensive investigation. Despite the known associations between these factors, only limited research specifically analyzing the tongue microbiota in children with MB undergoing orthodontic treatment is available [13]. We hypothesized that tongue microbiota in children with MB differs from that in children with NB. We have also previously observed changes in the quantity of specific oral bacteria and candidas in the gingival crevicular fluid (GCF) and dental plaque of children undergoing orthodontic therapy [14]; in view of these results, we planned to investigate tongue microbiota also in relation to the absence/presence of orthodontic appliances. The presented study aimed to analyze the associations of microbiota of the tongue's dorsa in children with orthodontic anomalies who are, therefore, at high POSA risk, from the perspective of their breathing pattern (MB/NB) before/during treatment with orthodontic appliance.

## Methods

### Design and clinical examination

In this pilot prospective case–control study, a meticulous methodology was employed to investigate the oral microbiota in a specific group of 30 children aged 7–12 years from the Czech Republic. The sample size of 30 children was determined based on prior studies examining microbiome changes in response to orthodontic therapy, which suggested that a sample of this size would be sufficient to detect significant differences in microbiome composition [14–17].

**Fig. 1:**
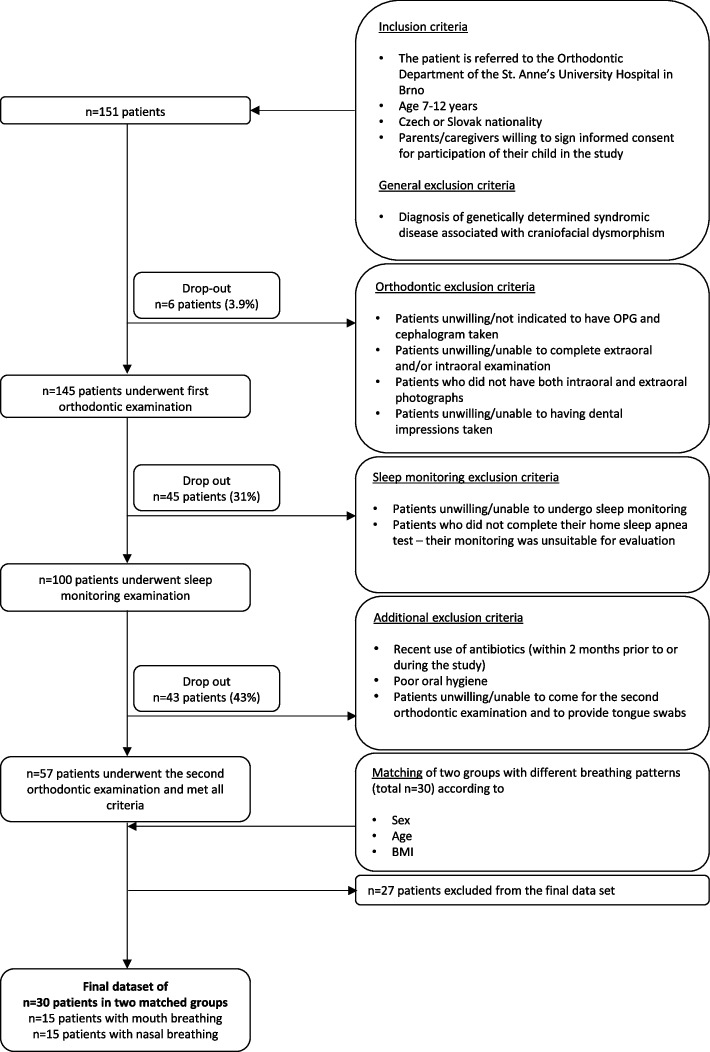
Flowchart with inclusion and exclusion criteria and matching of children for the study. n, number of patients; BMI, body mass index

All children were selected from the pool of patients from the Orthodontic Department of the Clinic of Stomatology, St. Anne’s University Hospital, Brno, Czech Republic. Our primary inclusion criteria were age 7 to 12 years, Czech or Slovak nationality, and parental consent for study participation. The exclusion criteria were (i) diagnosis of a genetically determined syndromic disease associated with craniofacial dysmorphism, such as Down syndrome or the Pierre-Robin sequence, criteria related to ii) orthodontic examination and iii) sleep monitoring, and iv) failure to undergo a repeated orthodontic examination with sample collection after approx. six-month therapy by orthodontic appliance, see Fig. 1 for more details. Additional exclusion criteria applied after orthodontic examination were v) recent use of antibiotics (within 2 months prior to and during the study) and/or vi) poor oral hygiene contraindicating the orthodontic treatment. Before the beginning of the orthodontic treatment, all patients received thorough instruction on proper oral hygiene from the dentist and, consequently, the level of oral hygiene was carefully evaluated at each follow-up to maintain the maximum homogeneity within the study group. If a patient's oral hygiene deteriorated during the study, he/she was referred to a dental hygienist for additional training and guidance.

From the pool of participants (57 meeting all inclusion and exclusion criteria, see flowchart in Fig. 1), 15 children with MB (cases) and 15 children with NB (controls) were selected and matched according to their age, sex, and BMI. The matching of MB and NB groups by these characteristics further ensured that the sample was adequate for the comparisons made.

The complex orthodontic examination included the extraoral and intraoral examination, photographs, dental impressions, and X-rays (cephalogram and orthopantomogram). Cephalometric analysis was conducted using lateral cephalograms (X-ray images taken in a standardized way (CS 9300, Carestream Dental, USA). Key landmarks included the Sella, Nasion, A-Point, B-Point, and Mandibular line (ML), which were used to identify the skeletal class (based on the ANB angle), mandibular positioning, and mandibular growth pattern (SN-ML angle). During this initial examination, we recorded not only the presence of orthodontic anomalies (specifically focusing on the anomalies that have been previously associated with a higher risk of airway narrowing and nocturnal breathing problems, such as maxillary constriction, increased overjet (> 3 mm), crowding (> 3 mm), skeletal class II – ANB > 5°, hyperdivergent skeletal pattern – SN-ML > 36°) [18], but also the overall habitus of the patient (e.g., adenoid facies), their BMI (BMI classification was based on the patients’ z-scores, which were calculated using Epi Info TM software (Centers for Disease Control and Prevention, CDC; in Atlanta, Georgia, USA), following WHO guidelines 2007 for children and adolescents (5–19 years) [19] and their breathing pattern. The preference for MB or NB was determined from full patient history taken from the parent of the patient, visual assessment of the lip posture, home sleep apnea monitoring, and clinical examination of the nasal movements. The visual assessment of lip posture was conducted with the patient in a relaxed, upright position. The lips were observed at rest to determine if they were naturally sealed (indicative of NB) or if they remained apart (suggestive of MB). This assessment was done repeatedly to account for any transient changes due to nervousness or temporary discomfort.

For the clinical examination of nasal movements, the patient was asked to maintain quiet, normal breathing while seated. The examiner observed the symmetry and amplitude of the nasal flaring with each breath. Particular attention was paid to any signs of nasal obstruction, such as reduced or asymmetric nasal movement, which might indicate compromised nasal airflow. These observations were used in conjunction with patient history and parental reports to classify breathing preferences accurately. All children were also referred for an ear, nose, and throat (ENT) examination for the endoscopic examination of the nose and nasopharynx (grade of nasopharyngeal tonsils, grade of palatine tonsils); however, only 16 patients were willing to undergo this examination, mainly due to the challenges posed by the COVID-19 pandemic. However, all patients underwent a comprehensive home sleep apnea examination using the Alice OneNight device, Philips Respironics (Murrysville, USA). This device monitored several key parameters, including snoring, which is crucial for evaluating nocturnal MB. The quality of their sleep-breathing and the risk of POSA was established based on the analysis of data obtained from the home sleep apnea monitoring using the scoring recommended for children under 12 years [20], apnea–hypopnea index (AHI) was determined.

The preparation for sample collection required stringent standardization, with participants and their parents receiving clear instructions not to eat, chew chewing gums, drink anything besides water, or perform any oral hygiene for 1 h before the procedure. The collection procedure was scheduled at two distinct intervals: before the beginning of the orthodontic treatment (timepoint M0) and approx. six months into the orthodontic therapy (timepoint M6).

The information about the sampling and analysis of oral candidas and bacteriome, including the process of candida´s analysis by MALDI-TOF mass spectrometry, DNA extraction from tongue swabs (*n* = 60) and negative controls (*n* = 10, DNA-free water, NCs) and 16S rRNA amplicon sequencing, as well as all the bioinformatic and statistical analysis, is closely described in the Supplement.

## Results

**Fig. 2:**
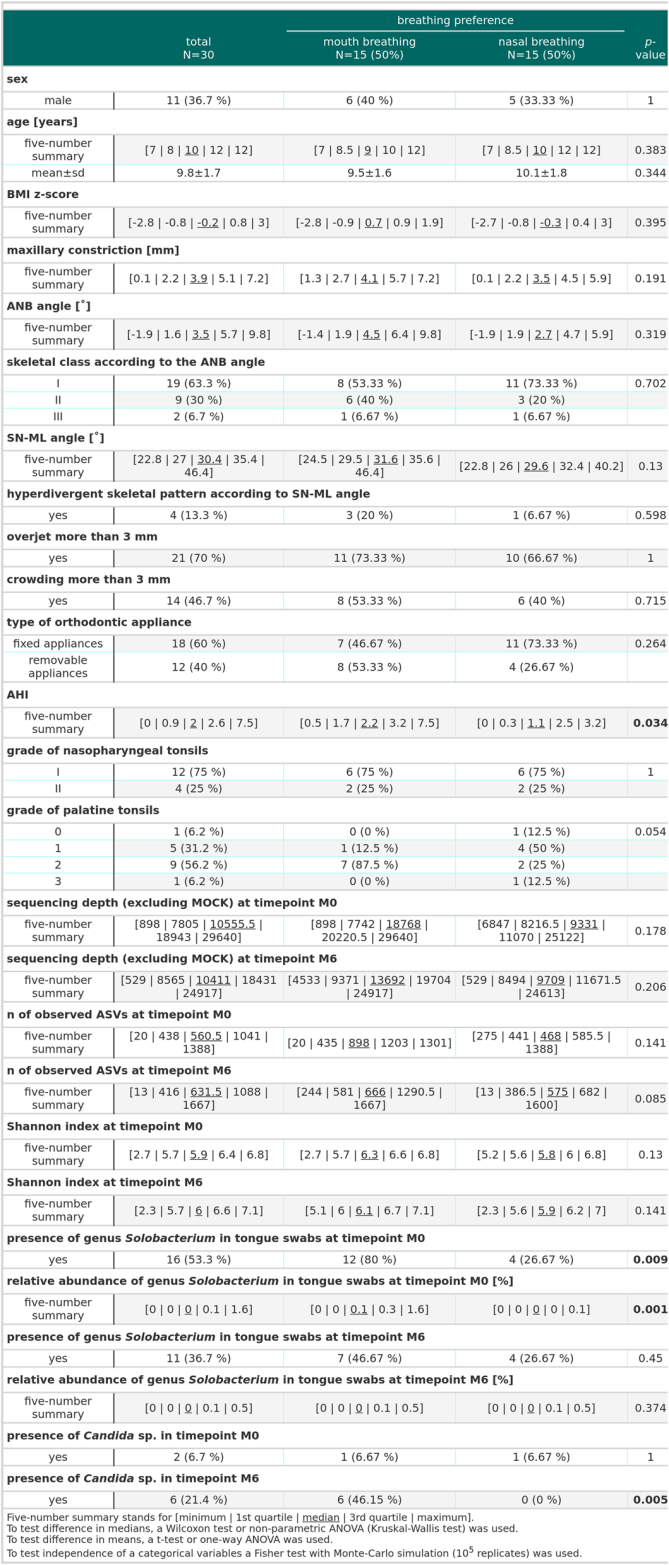
Demographic data and experimental results for studied children (*n* = 30) stratified according to their breathing preference. n, number of patients; SD, standard deviation; BMI, body mass index; ANB angle, angle between the points A, nasion and B; AHI, apnea–hypopnea index; M0, before orthodontic treatment; M6, approx. six months into the orthodontic therapy; MOCK, spiked commercial bacterial community; hyperdivergent skeletal pattern – SN-ML > 36°; skeletal class I, according to the ANB angle [-1°, 5°]; skeletal class II, according to the ANB angle > 5°; skeletal class III, according to the ANB angle < -1°

Distributions of the presence of the examined orthodontic characteristics (maxillary constriction, skeletal class according to the ANB angle, hyperdivergent skeletal pattern according to SN-ML angle, overjet, crowding, grade of nasopharyngeal tonsils, grade of palatine tonsils) were similar between studied groups (*p* > 0.05 for all). Also, patients with removable and fixed appliances were evenly distributed among groups of MB and NB (*p* > 0.05). The median AHI was found to be higher in the MB group than in the NB group (2.2 vs. 1.1, respectively, Fisher test, *p* = 0.034), see Fig. 2. None of the children in our study group had AHI higher than 10, thus a risk of severe POSA.

*Candida* sp. was found only in one patient in each group before the orthodontic treatment. However, during the orthodontic treatment (timepoint M6), *Candida* sp. were identified in six children with MB and in none of the children with NB (Fisher test, *p* = 0.012), see Fig. 2. Predominantly, *Candida albicans* was identified, although in one case, it cooccurred together with *Candida dubliniensis*, and in another case with *Candida lusitaniae*.

After quality filtering, chimeras removal and discarding MOCK (spiked commercial mock community, see Supplement) reads, 781,126 reads (five number summary: 113, 7012, 9,659, 18,306, 29,640) originated from patients’ samples and 13,219 reads (five number summary 113, 116, 1,258.5, 2,120, 3.391) from NCs.

**Fig. 3:**
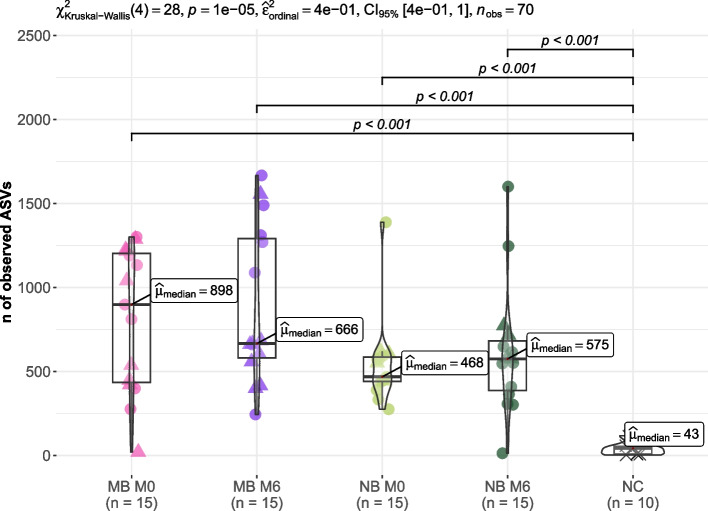
Number of ASVs of tongue bacteriomes in children (*n* = 30) stratified according to their breathing preference. ASVs, amplicon sequence variants; M0, before orthodontic treatment; M6, approx. six months into the orthodontic therapy; MB, mouth breathing preference; NB, nasal breathing preference; NC, negative extraction control (DNA-free water)

**Fig. 4:**
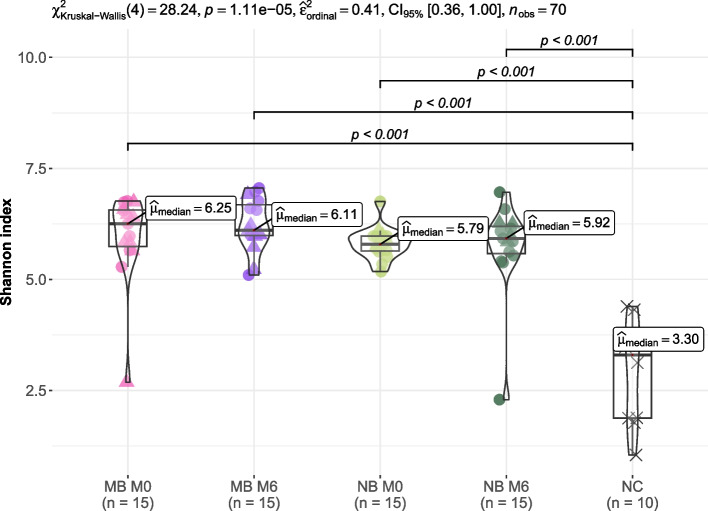
Shannon indeces of tongue bacteriomes in children (*n* = 30) stratified according to their breathing preference. M0, before orthodontic treatment; M6, approx. six months into the orthodontic therapy; MB, mouth breathing preference; NB, nasal breathing preference; NC, negative extraction control (DNA-free water)

No significant differences in alpha diversities (number of observed amplicon sequence variants – ASVs and Shannon index) of bacterial genera were detected between the MB and NB groups before or during the orthodontic treatment (*p* > 0.05 for all). However, tongue bacteriomes from each study group were significantly more diverse in the number of ASVs than NCs and Shannon indeces were also higher than in NCs (*p* < 0.001 for all), see Figs. 3 and 4, respectively.

**Fig. 5:**
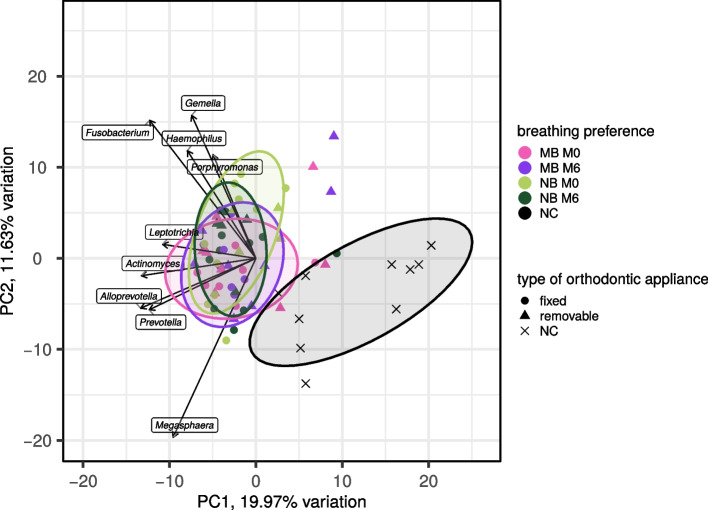
PCA of tongue bacteriomes in children (*n* = 30) stratified according to their breathing preference. M0, before orthodontic treatment; M6, approx. six months into the orthodontic therapy; MB, mouth breathing preference; NB, nasal breathing preference; NC, negative extraction control (DNA-free water); PCA, principal component analysis

**Fig. 6:**
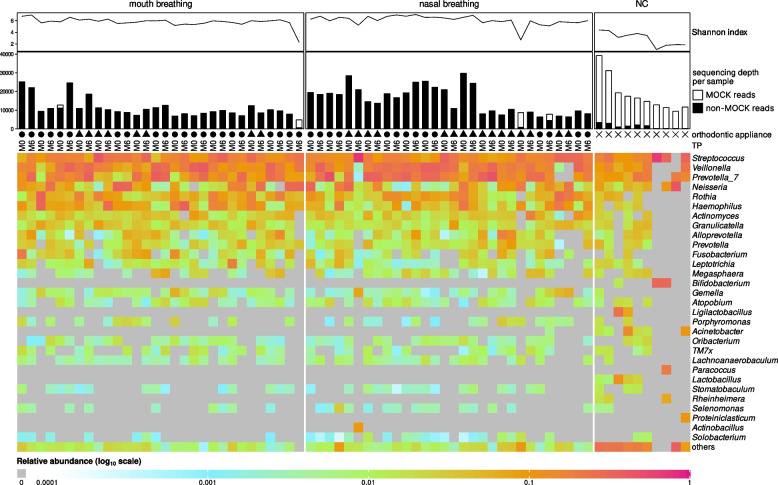
Heatmap of tongue bacteriome characteristics in studied children (*n* = 30). M0, before orthodontic treatment; M6, approx. six months into the orthodontic therapy; MB, mouth breathing preference; MOCK, spiked commercial bacterial community; NB, nasal breathing preference; NC, negative extraction control (DNA-free water)

Similarly, as in the case of alpha diversity principal component analysis (PCA) revealed no apparent differences between the timepoints, see Fig. 5. *Streptococcus, Prevotella 7, Veilonella, Neisseria, Rothia*, and *Haemophillus* were the most abundant genera, see the heatmap in Fig. 6. However, these bacterial genera were present also in NCs. Eleven bacterial genera were found exclusively in NCs (*Flavobacterium*, *Acinetobacter*, *Ligilactobacillus*, *Lactobacillus*, *Chlamydiaceae*, *Bifidobacterium*, *Paracoccus*, *Proteiniclasticum*, *Faecalibacterium*, *Oligoflexaceae*, *Butyricicoccus*).

**Fig. 7:**
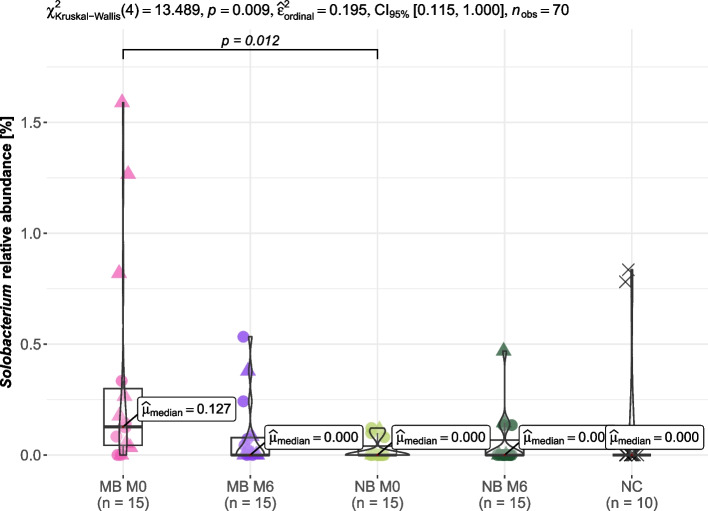
*Solobacterium* in tongue swabs from children (*n* = 30) stratified according to their breathing preference. M0, before orthodontic treatment; M6, approx. six months into the orthodontic therapy; MB, mouth breathing preference; NB, nasal breathing preference; NC, negative extraction control (DNA-free water)

At the level of genera, there were almost no differences in relative abundances of individual genera among patient groups and time points. However, before the start of the orthodontic therapy (timepoint M0), we observed a significantly higher relative abundance of the genus *Solobacterium* in children with MB than in children with NB (*q* = 0.012, Dunn's test), see Fig. 7. In line with this, the presence of genus *Solobacterium* in tongue swabs was significantly more common in children with MB than in those with NB before the initiation of orthodontic treatment (i.e., at timepoint M0, twelve vs. four children, respectively, *p* = 0.009), see Fig. 2.

To investigate whether the type of orthodontic appliances might have represented a confounding factor for the evaluation at the timepoint M6, we have performed additional analyses in the subset of patients with fixed appliances. Results of this subanalysis were consistent with those obtained for the entire study group, albeit statistical significance was not achieved for relative abundance of genus *Solobacterium* due to a smaller number of individuals (18 patients only) despite the presence of a clear trend.

In addition, we also analyzed whether the type of orthodontic appliance had any effect on presence of oral candidas and tongue bacteriome characteristics after approx. the six-month exposure but revealed no significant differences in the presence of oral candidas or bacteriome characteristics between patients with fixed and removable orthodontic appliances, see the Supplement for more details.

## Discussion

In the present study, we aimed to analyze the changes in tongue bacteriome in patients undergoing treatment with orthodontic appliances in association with mouth or nose breathing patterns and POSA. Our decision to use tongue swabs as a matrix of interest was driven by several reasons. Although unstimulated saliva is considered best matrix for the description of the microbiome in the oral environment, its collection is lengthy and, therefore, (especially in smaller children) difficult. Stimulated saliva, on the other hand, may not ascertain good representation of oral environment as the saliva does not get in touch with the entire oral environment [21]. Tongue swabs have been utilized in similar research and, besides the ease of collection, have been shown to be particularly relevant to conditions such as POSA from a pathophysiological perspective [22, 23]. In their 2022 study, Lu et al. also emphasized the benefits of tongue swabs and the difficulties associated with saliva collection [24].

The differences in bacterial diversity and composition between children with orthodontic anomalies undergoing orthodontic treatment were investigated according to their breathing preferences. The initial results of this study demonstrated that while no broad impacts of MB on oral bacteriome diversity were observed, there was a significant association between MB and the presence/higher relative abundance of the genus *Solobacterium*, suggesting a risk of halitosis linked to this breathing pattern [25]. Our results partially reflect previous findings regarding the differences in bacteriome composition between patients with MB and with NB [26, 27]. Since our study group did not include children at high risk of POSA, (i.e. those with AHI higher than 10), we did not conduct a bacteriome analysis in relation to this parameter.

Studies on microbial diversity in children with POSA have noted a significant difference in beta diversity; *Haemophilus, Fusobacterium*, and *Porphyromonas* were found in higher abundances in samples collected from adenoids and tonsils of patients with POSA than in controls [8, 13]. POSA also affects the microbiota composition of the buccal mucosa, altering levels of Firmicutes, Proteobacteria, Bacteroidetes, Fusobacteria, and Actinobacteria [27].

MB is often found in children with POSA but also in otherwise healthy subjects, further influencing the oral and nasopharyngeal microbiota, enriching bacteria with pathogenic potential, such as *Acinetobacter* sp. in supragingival plaque, *Neisseria* sp. in unstimulated saliva, *Streptococcus pneumoniae* in the pharynx, and *Stenotrophomonas* sp. in the nostrils [8]. MB has been also linked to chronic gingival inflammation and higher plaque index [26]. The tonsillar microbiota in patients with POSA differs, with a greater presence of genera such as *Porphyromonas, Moraxella*, and *Corynebacteria* in patients with POSA and adenotonsillitis and higher prevalence of *Leptotrichia, Campolybacter* or *Paludibacter* in children with POSA and adenotonsillar hypertrophy [28]. The surfaces of the adenoids and palatine tonsils harbor bacterial growth that may contribute to mucosal infection and influence POSA or recurrent tonsillitis [29]. Differences in abundance include higher abundances of *Parvimonas, Prevotella,* and *Treponema* in patients with recurrent tonsillitis, and *Haemophilus* and *Capnocytophaga* in patients with POSA, with a predominance of Proteobacteria and Firmicutes in sleep-disordered breathing [29].

The oral microbiome houses hundreds of microbial species with a dominance of Firmicutes, Bacteroidetes, Proteobacteria, Actinobacteria, Spirochaetes, and Fusobacteria [30]. Disruption of homeostasis in the oral microbiota can lead to a wide range of problems, including caries, gum inflammations, aphthae, halitosis, etc. [31]. Halitosis, or bad breath, is caused by volatile compounds produced mainly by anaerobic bacteria, such as sulfur compounds, ketones, sulfides, aldehydes, and amines. The tongue with its papillae and anaerobic sites offers a suitable environment to anaerobic bacteria causing halitosis [31]. In healthy subjects without halitosis, *Streptococcus salivarius* is the predominant species; in patients with halitosis, however, it was typically absent and the microbiota differed from healthy patients, with *Solobacterium moorei*, *Atopobium parvulum*, *Eubacterium sulci*, and *Fusobacterium periodonticum* showing the strongest associations with halitosis [32].

The higher relative abundance of genus *Solobacterium* in children with MB compared to children with NB is a significant result of our research. *Solobacterium* has been previously associated with halitosis both in children and adults [25, 33]. It thrives in anaerobic conditions, such as those found on the dorsum of the tongue or in the periodontal pockets, and is known for its sulfurous metabolic byproducts that contribute to bad breath [25, 32–34]. MB can often lead to dry mouth, known also as xerostomia, disrupts the natural balance of the oral environment [35]. This imbalance may result in an overgrowth of anaerobic bacteria, such as *S. moorei*, which may further intensify the problem of bad breath as it creates a favorable environment for these bacteria to decompose cellular debris and proteins, leading to the production of malodorous compounds [25, 33, 34]. Thus, MB may not only contribute to the conditions that favor the growth of *Solobacterium,* but can also intensify the symptoms of halitosis that the bacterium causes. This finding not only strengthens the link between specific bacterial genera and MB but also implies a potential pathophysiological role for *Solobacterium* in mouth breathers.

Multiple studies reported changes in the oral microflora during orthodontic therapy; however, most of them investigated saliva or dental plaque samples [15, 17]. In our recently published study, we emphasized the importance of the complex perspective in the research of the dynamics of the oral ecosystem [14]. We found that in patients whose plaque index deteriorated in the medium term after the bonding of the appliances (before the end of the 7th month of the treatment), the probability of finding any of the seven selected periodontal bacterial strains combined with oral candidas in GCF/dental plaque samples was 10 times higher than before their orthodontic therapy [14]. In line with this, the presence of *Candida* sp. was associated with exposure to six-month wearing of orthodontic appliance in children with MB in the current study. Tongue coating by *Candida* sp. is also considered as a risk factor of halitosis, as evidenced by the correlation between volatile sulfur compound levels and *Candida* sp. presence [36]. The study by Koga et al. also found higher concentration of methyl mercaptan, a key contributor to halitosis, in subjects with a strong positivity for *C. albicans* or with multiple *Candida* sp. [36].

Our study contributes to the field by highlighting the tongue as a critical site for the identification of microbial shifts, particularly in relation to MB and halitosis, in our study brought a novel perspective to the research on oral microbiota dynamics in the context of breathing patterns and orthodontic interventions in children. Moreover, our findings provide new insights into the impact of orthodontic appliances on microbiota composition over time, complementing existing literature on microbiota changes during orthodontic treatment [14–17, 37, 38].

The main limitation of our study lies in the process of diagnosis of the MB. There are several approaches to diagnosing the mouth-breathing pattern, including mirror and/or water retention tests [39]. In our study, we have used visual assessment of the lip seal, clinical examination of the nasal movements, and, most importantly, complex medical history taken from the patient’s parent. The latter is often used for diagnosing mouth breathing [40]; however, Costa et al. stated that such investigation may be insufficient and that children should be referred for an ENT examination [41], which we weren’t able to achieve in all our patients (in particular because of the ongoing COVID-19 pandemic), even though they have been all referred to an ENT specialist. Still, we can consider our pilot study methodologically strong because we have matched groups with many clinical parameters observed and examined across various medical fields. Moreover, the conditions for sample collection were very strict and the samples were analyzed using state-of-the-art approaches. Additionally, the variability in the type of orthodontic appliances used could have introduced confounding factor, although our supplementary analyses suggest that this did not affect the results, see Supplement. Moreover, it is important to keep in mind what the main result of the presented study is, i.e., that the relative abundance of the genus *Solobacterium* on the tongue is associated with mouth breathing even before the use of the orthodontic appliance.

## Conclusions

In conclusion, while the overall alpha diversity of the tongue bacteriome was not significantly affected by breathing preference in our study, the detected association between MB and the increased presence/relative abundance of genus *Solobacterium* highlights the potential implications for oral health, particularly in managing halitosis. Moreover, the presence of *Candida* sp., which are also a risk factor of halitosis, was found associated with approx. six-month treatment by orthodontic appliance but only in children with MB. These findings underscore the need for further research into targeted therapeutic strategies in children with MB and orthodontic anomalies.

## Supplementary Information

Additional file 1: Supplement.

## Data Availability

The data for this study have been deposited in the European Nucleotide Archive (ENA) at EMBL-EBI under accession number PRJEB79347 (https://www.ebi.ac.uk/ena/browser/view/PRJEB79347).
