# Supplementary material for: Tongue microbiota in relation to the breathing preference in children undergoing orthodontic treatment
Source: BMC Oral Health. 2024 Oct 21;24:1259. doi: 10.1186/s12903-024-05062-3 (PMC11492670; doi:10.1186/s12903-024-05062-3)
Supplement: Supplementary file 1 — Additional file 1: Supplement. [file 12903_2024_5062_MOESM1_ESM.docx]

**Tongue microbiota in relation to the breathing preference in children undergoing orthodontic treatment**

***Supplement***

Zuzana Marincak Vrankova^1,2,3^, Petra Brenerova^1^, Lenka Bodokyova^1^, Jan Bohm^1^, Filip Ruzicka^4^, Petra Borilova Linhartova^1,2*^

^1^ RECETOX, Faculty of Science, Masaryk University, Kotlarska 2, Brno, Czech Republic

^2^ Clinic of Maxillofacial Surgery, University Hospital Brno, Czech Republic

^3^ Clinic of Stomatology, Institution Shared with St. Anne´s University Hospital, Faculty of Medicine, Masaryk University, Brno, Czech Republic

^4^ Clinic of Microbiology, Institution Shared with St. Anne ́s University Hospital, Faculty of Medicine, Masaryk University, Brno, Czech Republic

**Methods**

***Sampling and analysis of oral candidas***

In all these children, oral mucosa swabs (COPAN Italia, Microbiology swab Transystem^TM^) at timepoints M0 and M6 were taken for the identification of *Candida* sp. The samples were resuspended in 1 ml phosphate buffered saline (PBS) and homogenized by vortexing. Subsequently, 100 ml of the resultant homogenate was inoculated onto Sabouraud's 4% maltose agar (Merck KGaA, Darmstadt, Germany) and CHROMagar Candida (CHROMagar, Paris, France). After 48 hours of incubation at 37 °C, yeast growth was assessed. All isolates were identified by Matrix-assisted laser desorption/ionization-time of flight (mass spectrometry (MALDI-TOF MS) using the MALDI BioTyper system (Bruker Daltonics) and FlexControl 3.4 software (Bruker Daltonics) according to the manufacturer instructions. Mass spectra were processed using BioTyper 3.1 software (Bruker Daltonics). Analysis is described in detail in our previous study [1].

***Sampling for bacteriome analysis***

At timepoints M0 and M6, the collection procedure involved careful swabbing of the dorsum of the tongue with a cotton swab (FLOQSwabs, COPAN ITALIA SpA, Brescia, Italy), which was then placed in a sterile 1.5 ml screw cap tube. The samples were immediately stored in a freezer at -80°C.

***DNA extraction and 16S rRNA amplicon sequencing***

The DNA was extracted from 30 tongue swabs collected at the timepoint M0 and 30 paired tongue swabs collected at the timepoint M6, ten negative extraction controls (DNA-free water, NC), and from the bacterial MOCK community ZymoBIOMICS Spike-in Control I (High Microbial Load, ZymoResearch, USA). DNA extraction was performed using the QIAmp DNA Mini Kit (QAIGEN, Germany) with addition of lysozyme (20 mg/ml). Incubation was in progress for 1 hour at 37 °C, according to the manufacturer's instructions.

The extracted DNA was amplified by PCR using Q5 High-Fidelity 2X Master Mix (New England BioLabs, USA) and Illumina primers (Elisabeth Pharmacon, Czech Republic). Before amplification, the reaction mastermix was decontaminated using 8-methoxypsoralen (MOP-8). Further, DNA from bacterial |MOCK community was used for spinning samples as well as NC.

All samples were then cleaned up using magnetic beads SPRIselect (Beckman Coulter, Czech Republic). The sequencing library of pooled samples was prepared using KAPA HiFi™ HotStart ReadyMix (2X) (Roche, Switzerland) and Nextera XT Index (Illumina, USA). After the second clean-up, the final library was pooled and three quality controls were run: TapeStation measurement using High Sensitivity D1000 ScreenTape (Agilent, USA), fluorimetry measurement using Quant-iT™ dsDNA Assay Kits high sensitivity (ThermoFisher, USA), and specific qPCR measurement using KAPA Library Quantification Kits (Roche, Switzerland). The MiSeq Reagent Kit v3 (600-cycle) was used for bacteriome sequencing. Sequencing was conducted on the MiSeq™ System (Illumina, USA).

***Bioinformatic analysis of data from 16S rRNA amplicon sequencing***

Forward and reverse pair-end reads were demultiplexed, and barcodes and primers were trimmed using cutadapt (v4.2) [2] and a known set of sequencing primers. Quality profiling was done by fastQC (v0.11.5) [3] and DADA2 (v1.22) [4] algorithm. Trimming parameters were estimated with a median quality score drop below 20 and the minimum fraction of reads to retain was set to 0.75. A denoising algorithm with DADA2 was applied separately on forward and reverse reads that passed the quality and length filter (>50 bp) and trimming. Reads were merged using the fastq-join method. In the next step, chimeras were detected with the function removeBimeraDenovo in DADA2. Chimera sequences were subsequently excluded from the analysis, and an amplicon sequence variant (ASV) table was created. The table was filtered to remove unwanted mitochondrial and chloroplast taxa and a length filter (>100 bp) was applied. Default parameters recommended by nf-core/ampliseq (v2.6.1) [5] were used if not stated otherwise.

Taxonomy was assigned to each ASV based on SILVA (v138) [6] reference database using the algorithm RDP Naive Bayesian Classifier algorithm from DADA2 implementation. The BLAST (v2.10.0) [7] algorithm was used to identify the species, and all taxa with the maximum identity and minimum e-value were selected for each ASV from the NCBI 16S ribosomal RNA database (v2023-08). The alpha diversities for each sample were computed using QIIME2 (v2022.11.1). Picrust2 (v2.5.0) was employed to predict hypothetical abundances of KEGG orthologs in each sample and to summarize them into higher functional processes to obtain estimates of metabolic potential of the microbial communities.

***Statistical analysis***

All biostatistical analyses were performed using R version 4.1.2 (2021-11-01) [8].

Fisher test (categorical variables) and Kruskal-Wallis test (continuous variables) were used for the comparison of demographic and clinical characteristics, i.e. oral candidas, among the groups of interest. For clinical data, missing data from ENT examination (grade of nasopharyngeal tonsils and grade of palatine tonsils) were excluded from descriptive statistics.

Analyzing alpha diversity began by retaining samples with a minimum of 1000 reads, encompassing the bacterial MOCK community. Subsequently, mock community reads were excluded, and alpha diversity was calculated on the filtered dataset. Two diversity indices— Shannon index, and ASV count—were computed using the vegan (v2.6-4) [9]. Group distinctions were assessed using the nonparametric Kruskal-Wallis test, accompanied by post-hoc analyses involving paired and two-sample Wilcoxon tests. Visualization was facilitated by the ggplot2 (v3.4.3) [10], ggstatsplot (v0.12.0) [11], and ggsignif (v0.6.4).

For exploring beta diversity, a heatmap illustrating relative abundances (excluding bacterial MOCK community reads) was generated using the ComplexHeatmap (v2.10) [12] and seriation (v1.5.1) [13]. Principal Component Analysis (PCA), implemented through the PCAtools (v2.6.0) [14], was executed on CLR‑transformed data. Zero values were replaced with half of the smallest non-zero value in the dataset. In the PCA plot, the top 5% of loadings were displayed.

Subsequently, each bacterial genus underwent a Kruskal-Wallis test to evaluate differences in relative abundance among groups. Subsequent pairwise assessments encompassed both relative abundance and the presence of specific genera, employing Wilcoxon tests and proportion tests. To control for multiple comparisons, adjustments were applied using the Holm–Bonferroni method for tests within each genus batch.

**Results and Conclusions**

***Sub-analysis of tongue bacteriome in relation to breathing preference in patients with fixed orthodontic appliances only***

All patients who were indicated for treatment with removable orthodontic appliances were excluded from this sub-analysis and retained only those with fixed appliances (n=18). Among them, seven patients had a preference for mouth breathing and eleven for nasal breathing. These groups were compared using the same design as described in the main body of the article.

The results in this subset are consistent with the results of the entire cohort, which included also the patients with removable orthodontic appliances. In seven patients with MB, the numbers of observed ASVs were higher in timepoint M6 than in timepoint M0 (*p*=0.024), see Figure S1.

However, no difference in Shannon index between groups or time points was found in paired samples (*p*>0.05), as shown in Figure S2. Similarly, no differences were found in the composition of the bacteriome between NB and MB before or during the orthodontic therapy, although an insignificant trend towards a higher relative abundance of the genus *Solobacterium* in MB was observed prior to the orthodontic therapy initiation, as shown in Figure S3.

**
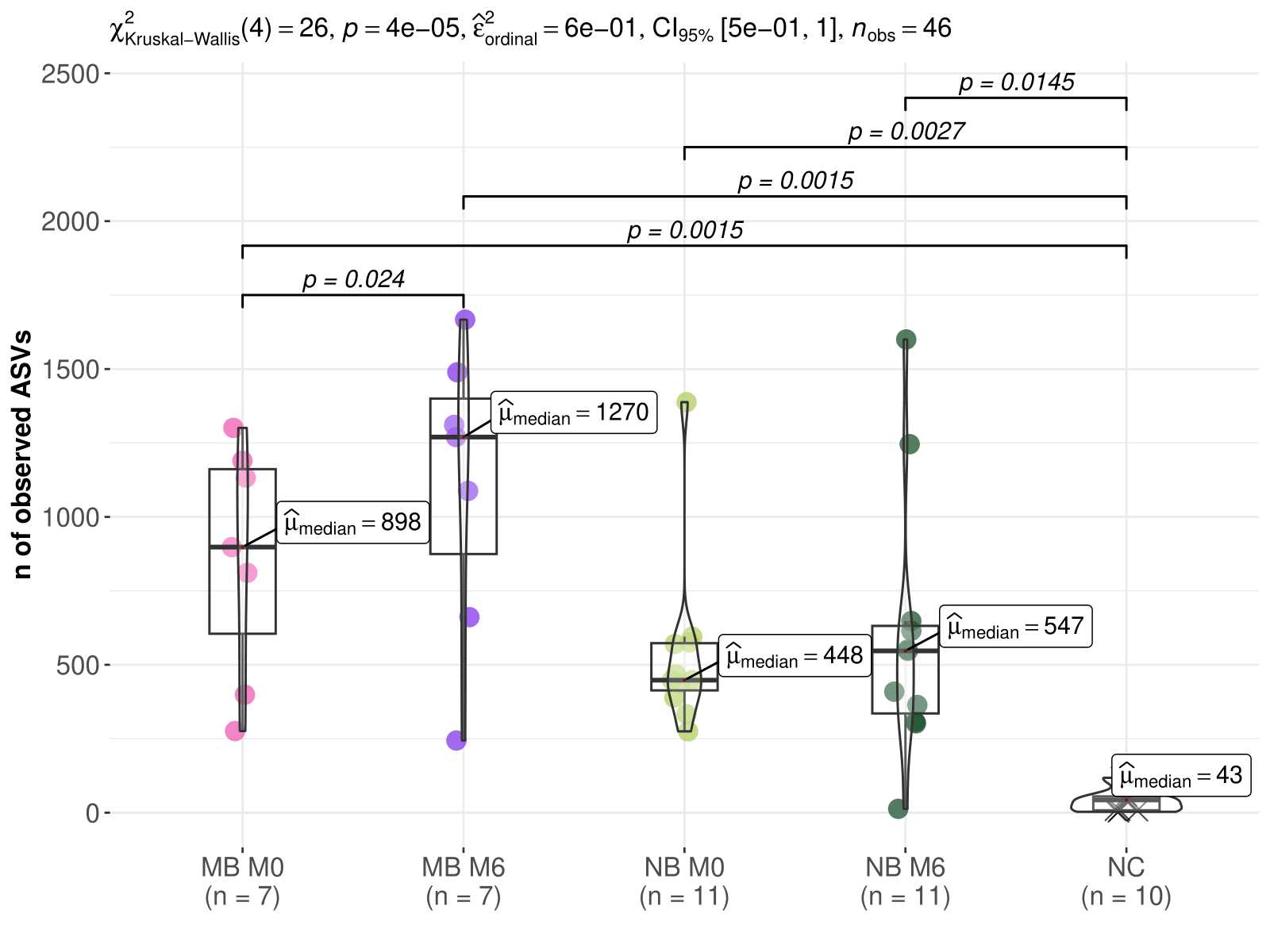
**

**Figure S1.** Number of ASVs of tongue bacteriomes in children (n=18) undergoing orthodontic treatment with fixed appliance stratified stratified according to their breathing preference

ASVs, amplicon sequence variants; M0, before orthodontic treatment; M6, approx. six months into the orthodontic therapy; MB, mouth breathing preference; NB, nasal breathing preference; NC, negative extraction control (DNA-free water)


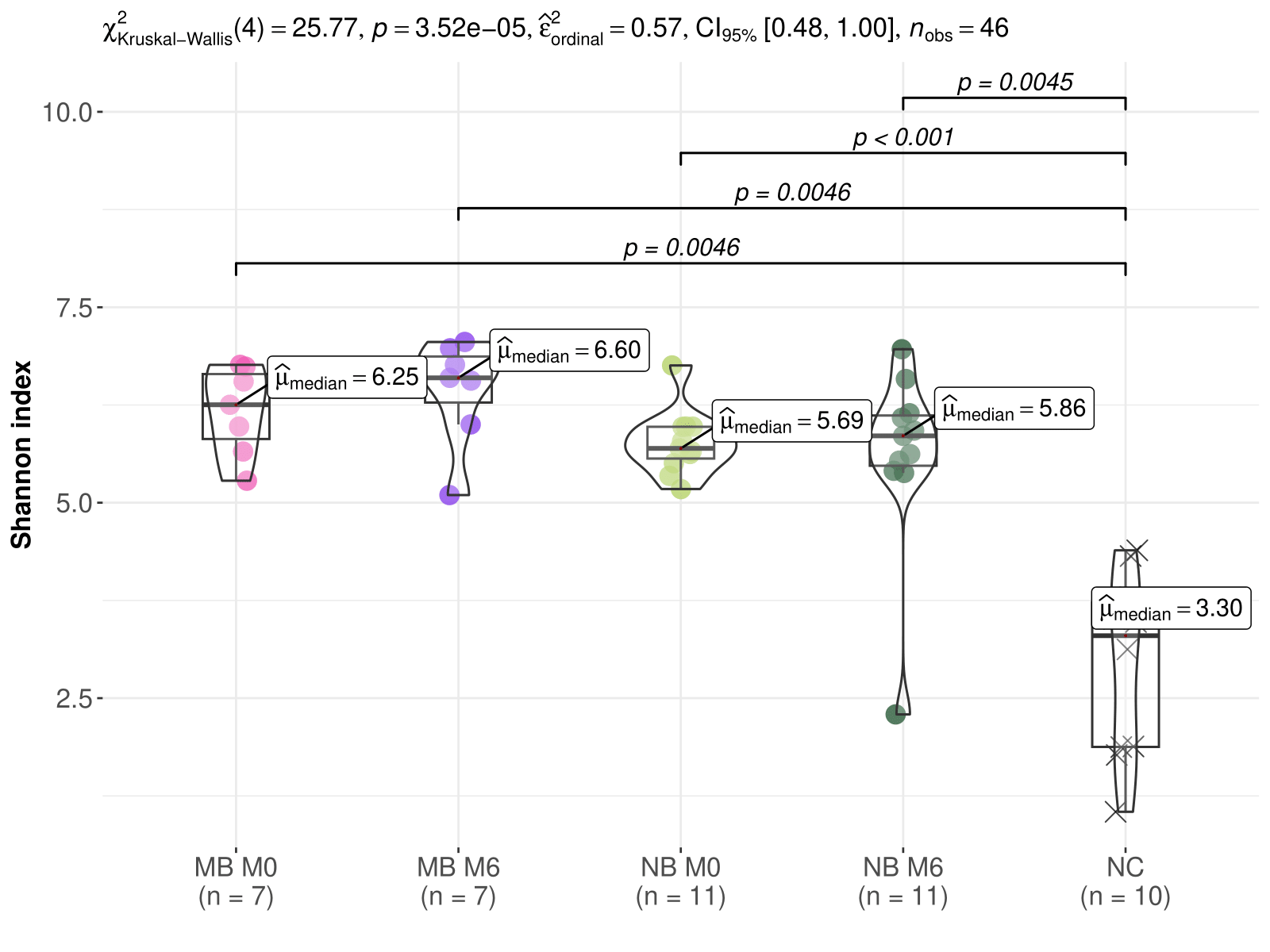
**Figure S2.** Shannon indeces of tongue bacteriomes in children (n=18) undergoing orthodontic treatment with fixed appliance stratified according to their breathing preference

M0, before orthodontic treatment; M6, approx. six months into the orthodontic therapy; MB, mouth breathing preference; NB, nasal breathing preference; NC, negative extraction control (DNA-free water)

**Figure S3.** Relative abundance
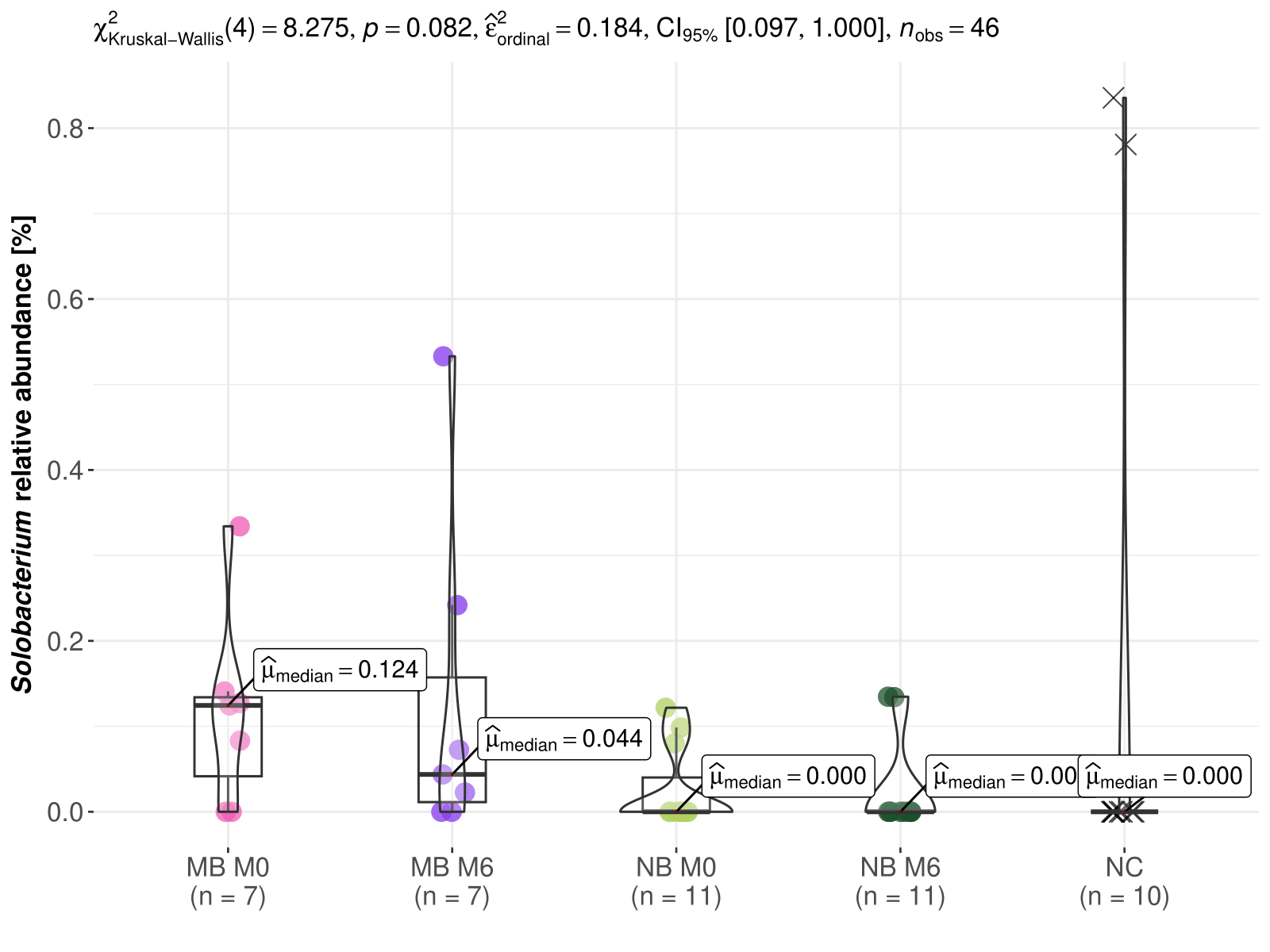
s of genus *Solobacterium* in tongue swabs from children (n=18) with orthodontic anomalies undergoing orthodontic treatment with fixed appliance stratified according to their breathing preference

M0, before orthodontic treatment; M6, during the treatment, approx. six months into the orthodontic therapy MB, mouth breathing preference; NB, nasal breathing preference; NC, negative extraction control (DNA-free water)

Unlike in the entire study group (30 patients), we did not achieve a statistically significant result for this bacterial genus in the group with fixed appliances only; however, this is rather due to the smaller number of participants as there was a clear trend consistent with the results for the entire study group.

***Results of analysis in all patients stratified according to orthodontic appliance type***

All 30 patients were stratified based on the type of orthodontic appliance they received (18 children with a fixed appliance, 12 children with a removable appliance,). We then performed paired analyses at the time points (a) before fitting of the orthodontic appliance and (b) approx. six months later.

The children with fixed appliances were older than those with removable appliances (*p*<0.01); no other significant differences were observed between the groups (*p*>0.05), as shown in Figure S4. No difference in the alpha diversity (in both ASVs and Shannon index) between groups or time points in paired samples (*p*>0.05) was found, as shown in Figure S5 and Figure S6. The PCA did not reveal any statistically significant differences between groups and time points, either, as shown in Figure S7. Figure S8, shows that the relative abundance of the genus *Solobacterium* is similar between time points and groups (*p*>0.05), as was the case for all other bacterial genera.


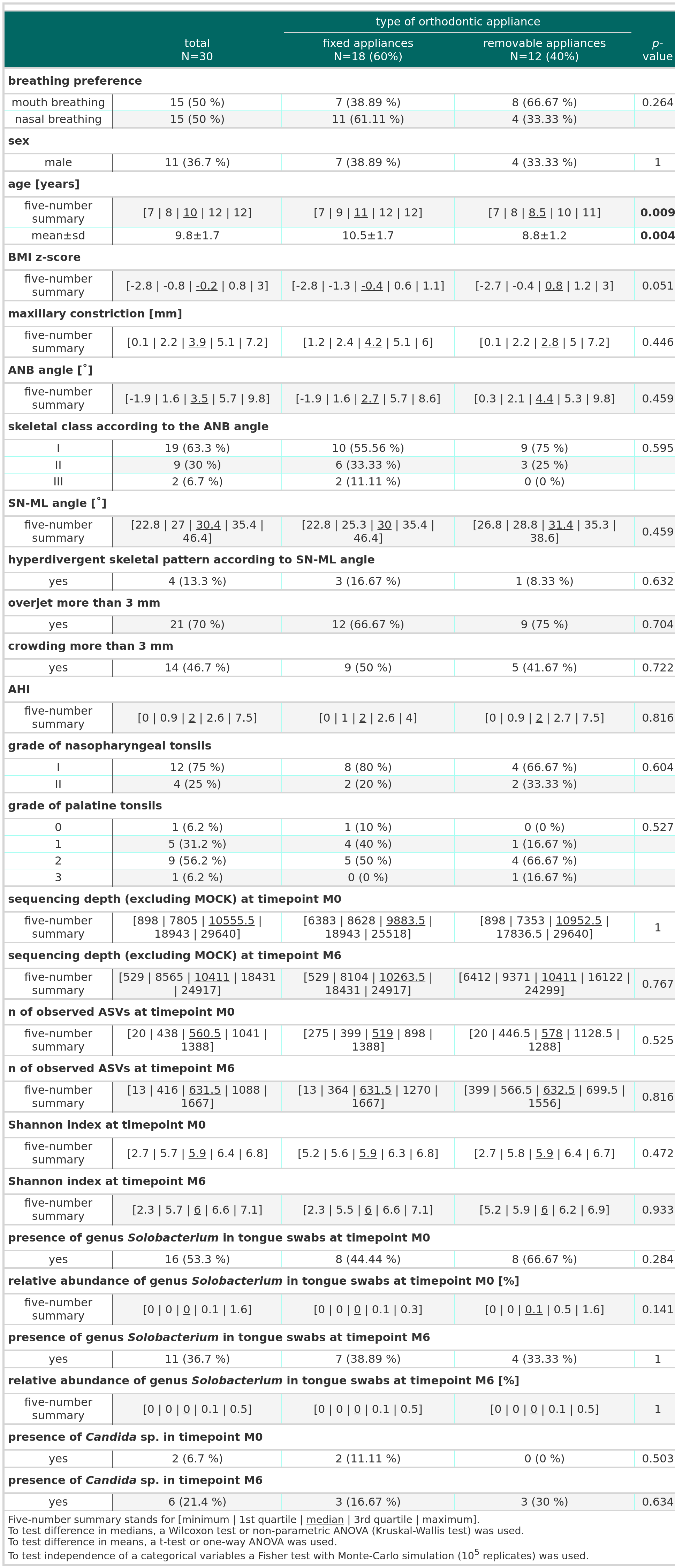


**Figure S4.** Demographic data for studied children (n=30) stratified according to the type of orthodontic appliance they received

N, number of patients; SD, standard deviation; BMI, body mass index; ANB angle, angle between the points A, nasion and B; AHI, apnea-hypopnea index; M0, before orthodontic treatment; M6, approx. six months into the orthodontic therapy; MOCK, spiked commercial bacterial community; hyperdivergent skeletal pattern – SN-ML >36°; skeletal class I, according to the ANB angle [-1°, 5°]; skeletal class II, according to the ANB angle >5°; skeletal class III, according to the ANB angle <-1°


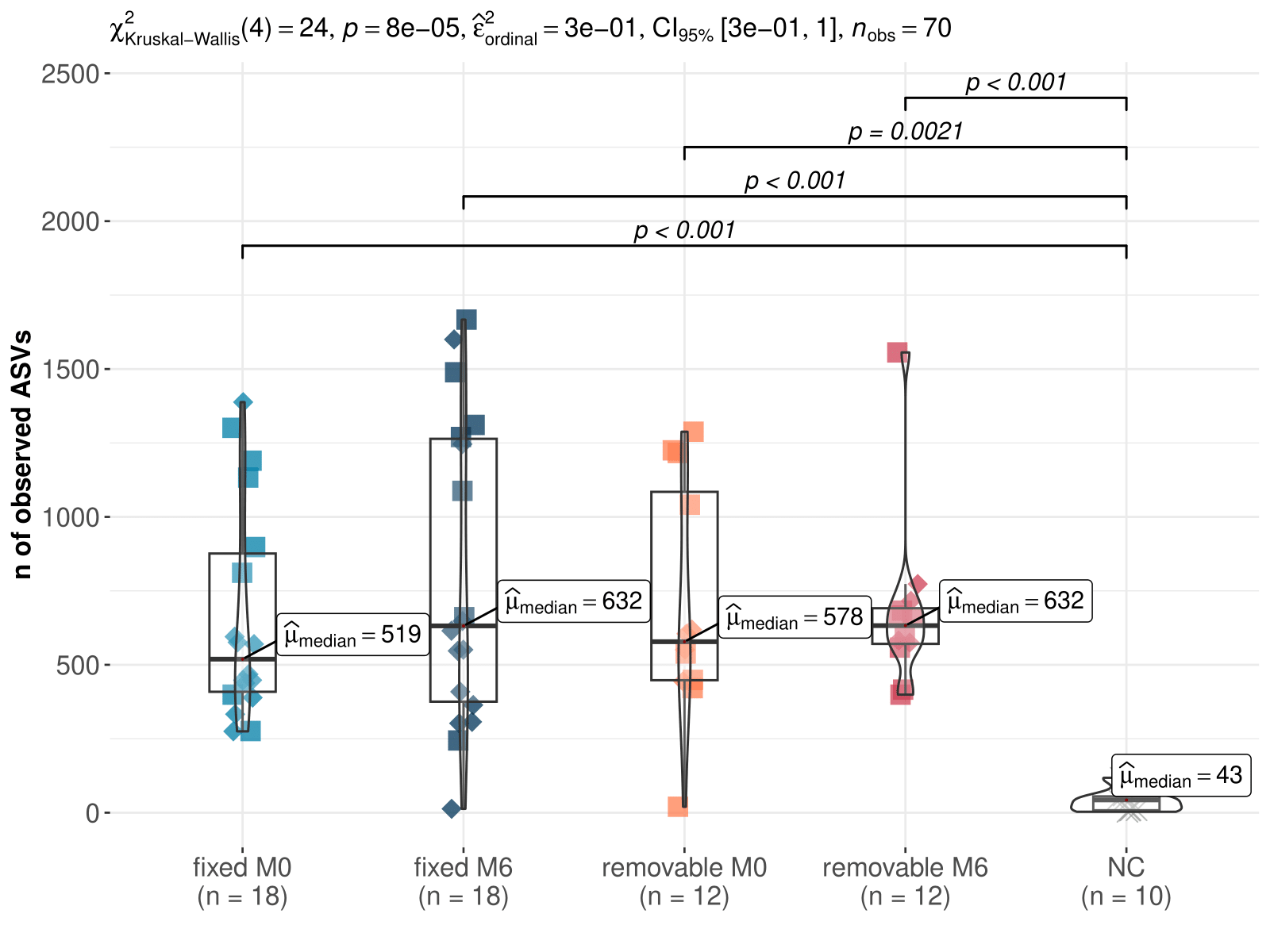


**Figure S5.** Number of ASVs of tongue bacteriomes in children (n=30) according to the type of orthodontic appliance they received

ASV, amplicon sequence variant; M0, before orthodontic treatment; M6, approx. six months into the orthodontic therapy; MB, mouth breathing preference; NB, nasal breathing preference; NC, negative extraction control (DNA-free water)


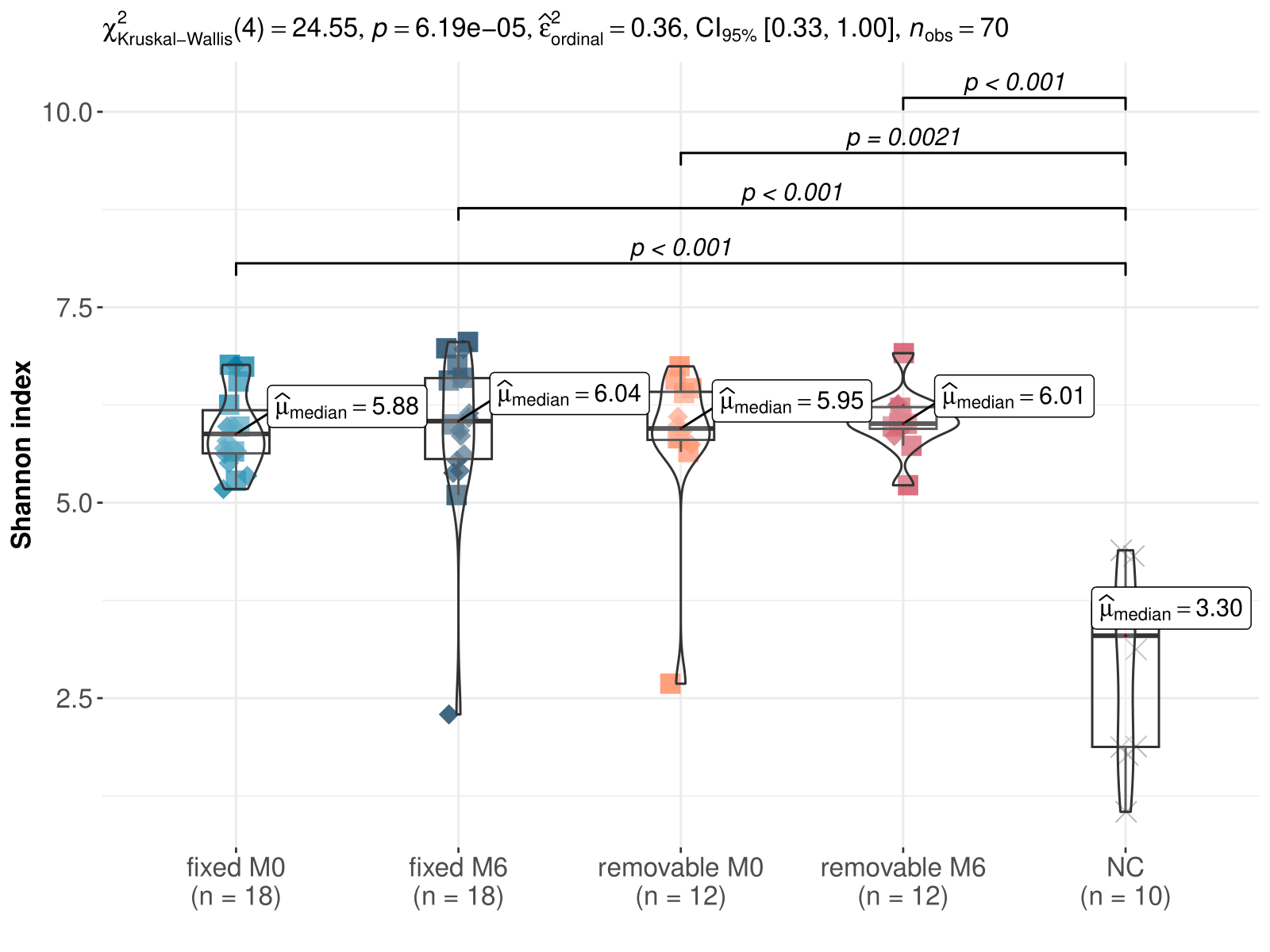


**Figure S6.** Shannon indeces of tongue bacteriomes in children (n=30) according to the type of orthodontic appliance they received

M0, before orthodontic treatment; M6, during the treatment, approx. six months into the orthodontic therapy; MB, mouth breathing preference; NB, nasal breathing preference; NC, negative extraction control (DNA-free water)


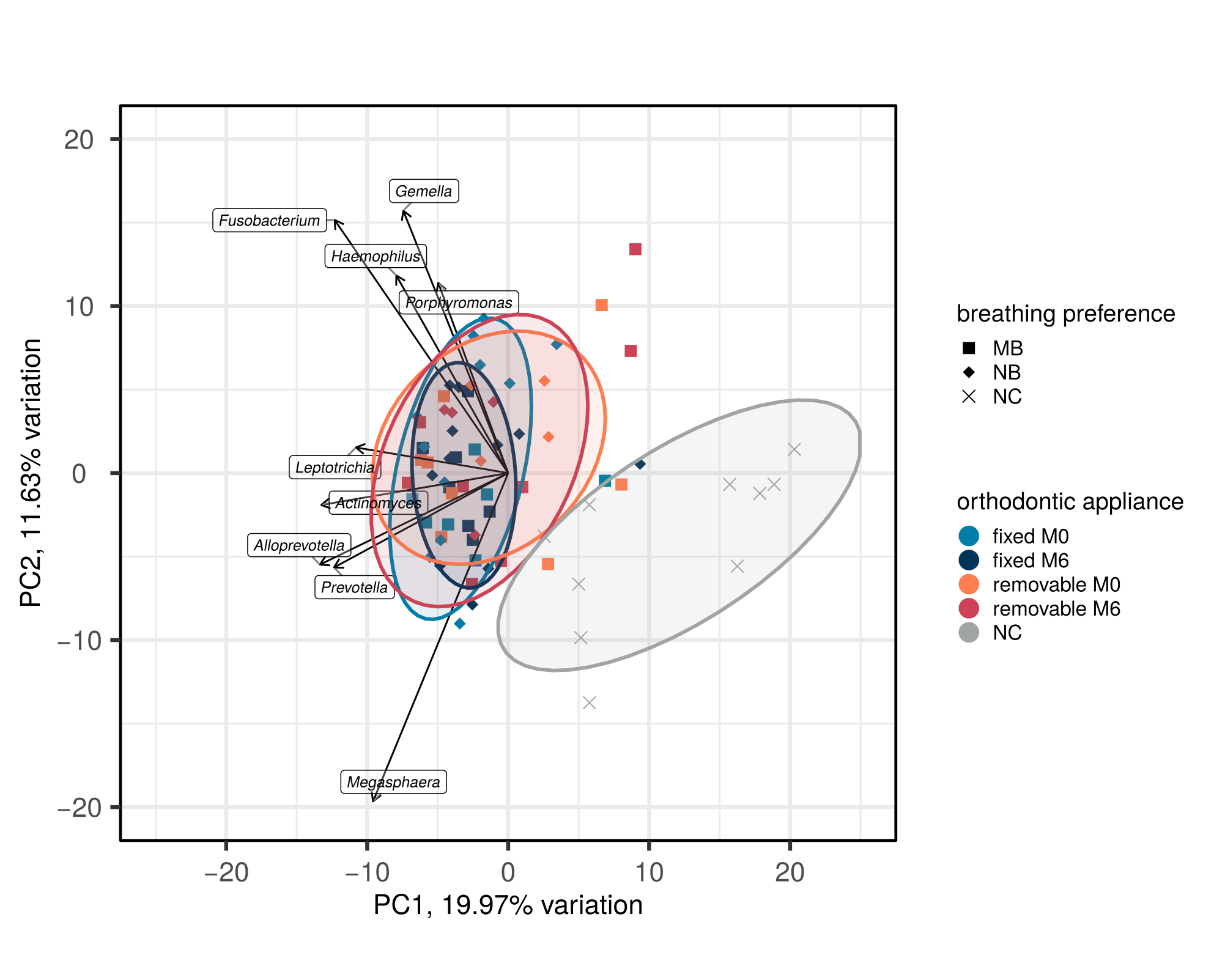


**Figure S7.** PCA of tongue bacteriomes in children (n=30) stratified according to the type of orthodontic appliance they received

M0, before orthodontic treatment; M6, approx. six months into the orthodontic therapy; MB, mouth breathing preference; NB, nasal breathing preference; NC, negative extraction control (DNA-free water); PCA, principal component analysis


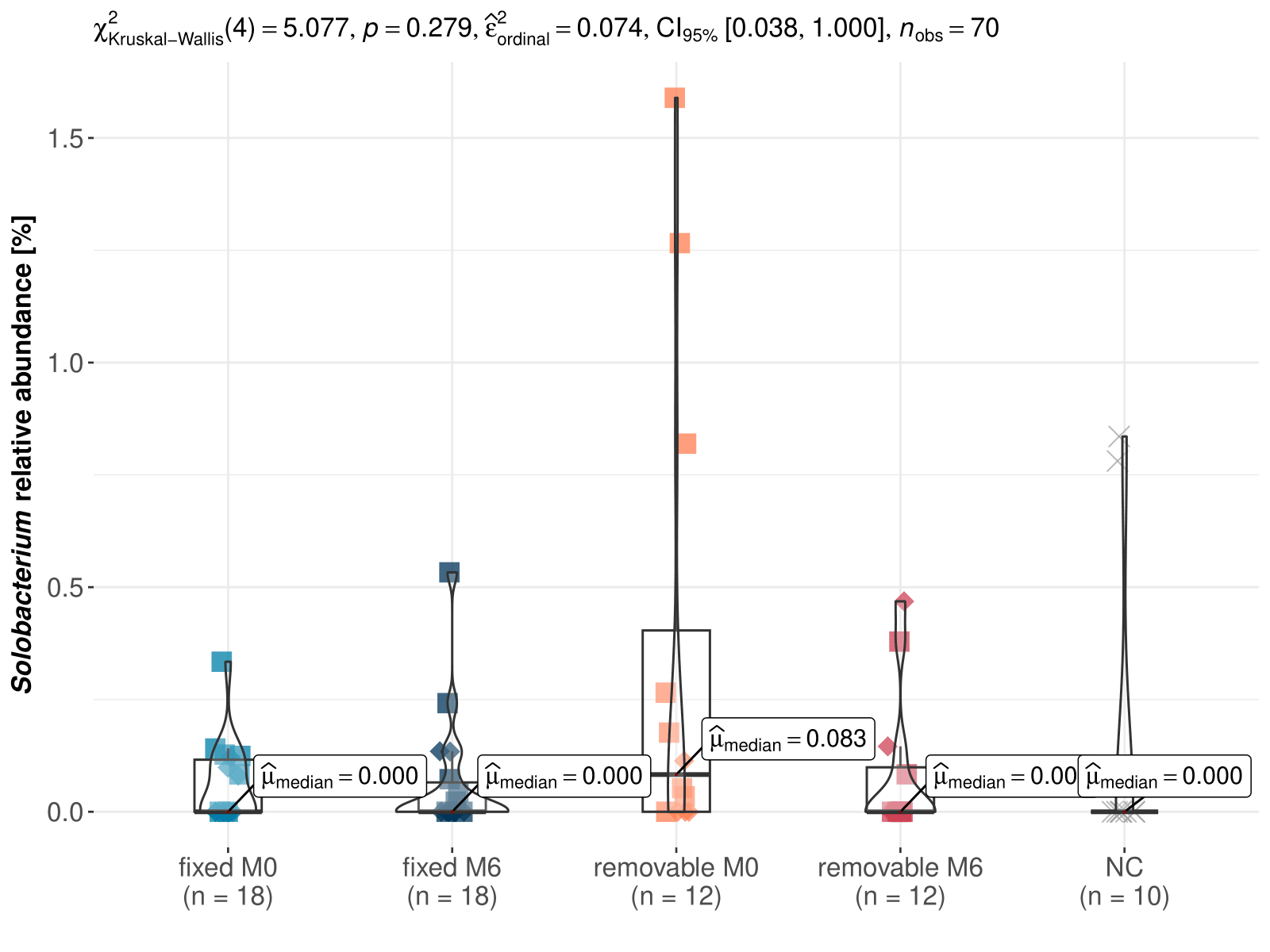


**Figure S8.** Relative abundances of genus *Solobacterium* in tongue swabs from children (n=30) stratified according to the type of orthodontic appliance they received

M0, before orthodontic treatment; M6, during the treatment, approx. six months into the orthodontic therapy MB, mouth breathing preference; NB, nasal breathing preference; NC, negative extraction control (DNA-free water)

The lack of differences between the groups of children treated with different orthodontic appliances may reflect their good education on oral hygiene and adherence to these recommendations.

**References**

1. Cvanova M, Ruzicka F, Kukletova M, et al. *Candida* species and selected behavioral factors co-associated with severe early childhood caries: Case-control study. *Front Cell Infect Microbiol*. 2022;12:943480. doi:10.3389/fcimb.2022.943480

2. Martin M. Cutadapt removes adapter sequences from high-throughput sequencing reads. *EMBnet journal*. 2011;17(1):10-12.

3. Andrews S. FastQC: a quality control tool for high throughput sequence data. Babraham Bioinformatics, Babraham Institute, Cambridge, United Kingdom; 2010.

4. Callahan BJ, McMurdie PJ, Rosen MJ, Han AW, Johnson AJA, Holmes SP. DADA2: High-resolution sample inference from Illumina amplicon data. *Nature methods*. 2016;13(7):581-583.

5. Straub D, Blackwell N, Langarica-Fuentes A, Peltzer A, Nahnsen S, Kleindienst S. Interpretations of environmental microbial community studies are biased by the selected 16S rRNA (gene) amplicon sequencing pipeline. *Front Microbiol*. 2020;11:550420.

6. Quast C, Pruesse E, Yilmaz P, et al. The SILVA ribosomal RNA gene database project: improved data processing and web-based tools. *Nucleic acids research*. 2012;41(D1):D590-D596.

7. Camacho C, Coulouris G, Avagyan V, et al. BLAST+: architecture and applications. *BMC Bioinformatics*. 2009;10:421. doi:10.1186/1471-2105-10-421

8. R Core Team A, Team RC. R: A language and environment for statistical computing. R Foundation for Statistical Computing, Vienna, Austria. 2012. 2022.

9. Jari Oksanen F, Friendly M, Kindt R, et al. Vegan: community ecology package. *R package version*. 2018;2(6)

10. Wickham H, Chang W, Wickham MH. Package ‘ggplot2’. *Create elegant data visualisations using the grammar of graphics Version*. 2016;2(1):1-189.

11. Patil I. Visualizations with statistical details: The'ggstatsplot'approach. *Journal of Open Source Software*. 2021;6(61):3167.

12. Gu Z, Eils R, Schlesner M. Complex heatmaps reveal patterns and correlations in multidimensional genomic data. *Bioinformatics*. 2016;32(18):2847-2849.

13. Hahsler M, Buchta C, Hornik K. seriation:Infrastructure for Ordering Objects Using Seriation. 2023. R package version 1.5.1. https://CRAN.R-project.org/package=seriation.

14. Blighe K, Lun A. PCAtools: Everything Principal Components Analysis. R Package Version 2.2. 0; 2020. 2021.
